# Small is Not Beautiful: A Critique of Antitrust Economics and Policy in Health Care

**DOI:** 10.1177/10775587261430879

**Published:** 2026-04-19

**Authors:** James C. Robinson

**Affiliations:** 1 University of California, Berkeley, USA

**Keywords:** integrated organization, managed competition, antitrust, vertical integration, horizontal integration

## Abstract

The dominant health policy perspective today appears to be that markets are over-consolidated and in need of anti-trust intervention. This commentary argues that the diagnosis is largely incorrect and the proposed treatment ineffective or, worse, effective but undesirable. The anti-trust ideal of a fragmented cottage industry of physician practices, hospitals, and other care organizations does not promise efficiency, even if the current industry configuration also is far from ideal. Many health care market are indeed competitive, and most are contestable, when one views provider organizations as multiproduct firms competing in overlapping geographic and customer markets. For example, many hospital systems compete in overlapping markets for primary care, specialty procedures, drug infusion, home health, subacute care, transplant surgery, telemedicine, population health management, and other services beyond traditional acute inpatient care. The anti-trust perspective should articulate its short-term and its long-term vision for the future of health care. What if it stopped all further mergers but left the status quo in place. What then? What if it not only stopped mergers but reversed them and brought us back to the health care industry of 1980? What then?

## Introduction

Today many health economists and policy analysts criticize large hospital-centered organizations that combine inpatient facilities, physician practices, ambulatory surgery, home health, and other services. Many more, perhaps, criticize publicly traded conglomerates that combine insurers, pharmacy benefit managers, physician clinics, and, indeed, almost all health services except hospital facilities. These critiques find some support in empirical research. Prices often are higher in local markets that feature few competitors, hospital-based infusion clinics charge higher drug prices than freestanding alternatives, and the subsidiaries of diversified organizations buy and sell among themselves. Antitrust enforcement often is prescribed to prevent mergers and acquisitions (M&A), regulation to prevent the private equity rollup of physician practices, and divestiture to unscramble the omelet that is United Health Group.

How far we have come. It was not too long ago, just a generation, when small was not so beautiful. The legacy health care system was a cottage industry of single-specialty physician practices and independent community hospitals, financed by insurers that paid fee-for-service to the doctors and cost-based reimbursement to the facilities. The empirical research of that era documented variability in procedure appropriateness and clinical outcomes, weak attention to preventive care and patient experience, and ever-rising prices and spending. The goal of that generation’s critics was system efficiency and innovation, and the prescription was organizational integration. Many health policy analysts advocated prepaid group practice, vertically integrated with health insurers, and horizontally integrated with hospital systems.

America duly set off on the path toward integrated health care organization. But now big is bad, or at least M&A to become big is bad, or at the very least M&A to become big enough to influence prices is bad.

## Integrated Organization in Health Care

Viewed against a status quo of fragmented firms and collusive professional practices, a health care system built around integrated organization offered great promise. In principle, prepaid multispecialty physician practices could assume clinical and financial responsibility for their patient populations. Hospitals could move from the center to the periphery of local health care communities, displaced by ambulatory and home-based care. Efficiency, quality, and innovation could be enhanced by competition among organizations, rather than among individual providers. The new vision was labeled the consumer choice health plan, then managed competition, then managed care, then value-based care.

Suffice it to say that the revolution never quite happened.

M&A often created slow-moving conglomerates that were more responsive to provider rather than patient preferences, suffered from internal culture clashes, insisted on fee-for-service payment, emphasized treatment over prevention, and kept hospital facilities at their core. Those that failed to diversify remained too small to bear the financial risk of capitation, too specialized to manage patients with complex comorbidities, and too weak to survive the fluctuation in revenues from budget-constrained payers. Reducing costs was hard, and so the integrated organizations often focused on increasing revenues.

## The Antitrust Critique

The stumbling process of organizational integration gave life to a different diagnosis and prescription for health system reform. The antitrust tradition in economics has always been skeptical of large organizations or at least of mergers that created organizations large enough to dominate local markets. The ‘Chicago’ variant focuses on market structure and pricing, balancing the virtues of efficiency with the vices of pricing power, whereas the ‘Brandeis’ variant takes a skeptical view of large organization per se. Economists working in the Chicago tradition note that most hospital markets exceed the consolidation thresholds established by the federal regulatory authorities while those working in the Brandeis tradition emphasize the sheer scale of health care firms that have diversified across products and services. Some economists acquiesce to large scale and market dominance if achieved through incremental growth, as that implies the firms are selling something consumers want to buy at prices they are willing to pay, while opposing growth through merger. Some denounce rollups that create larger organizations even if they do not achieve market dominance. Others denounce large firms even if they have grown without benefit of M&A.

### But How Well do the Antitrust Perspectives Diagnose the Ills of the Health Care Industry?

#### Are Current Health Care Markets Not Competitive?

Hospitals are multiproduct firms that sell services in overlapping markets. The geographic scope of the markets for emergency care, scheduled surgery, maternity, and organ replacement are very different. Primary care is local, but telemedicine has converted behavioral health into regional markets. Air transport has done the same for emergency medicine. Hospital outpatient departments compete with freestanding ambulatory surgery, diagnostic, rehabilitation, and urgent care centers. Many patients are willing to travel to a large regional facility in preference to a small local one. Some patients are willing to travel to other nations for cheaper or more culturally sensitive care. Some hospital systems define themselves as Centers of Excellence to compete for patients needing interventional cardiology, brain cancer care, and other complex services. Who has measured the structure for each of these markets?

#### Are Health Care Markets Not Contestable?

A market is deemed ‘contestable’ if entry barriers are low, even if there is a sole provider, since efforts by the incumbent to raise price above competitive levels quickly would draw in new competitors. For many health care services, barriers to entry are reasonably low and markets are reasonably contestable. Advances in technology are enabling services that formerly were provided in acute and inpatient settings to be offered in ambulatory settings and at home. Multi-market chains of ambulatory surgery centers, behavioral health providers, subacute facilities, and home health agencies can readily expand from one market to another if a revenue opportunity beckons. Entry barriers for inpatient hospital services are high, but diversified conglomerates can enter the market and restrict their offering to the most profitable services. Many hospitals lose money on traditional inpatient services and rely on ambulatory care, with its low barriers to entry, to help cover their fixed costs.

#### Do Health Care Markets Exhibit Monopolistic Pricing?

Hospitals are price takers rather than price makers with respect to their largest payers, the traditional Medicare and Medicaid programs, and even the Medicare and Medicaid managed care plans that must negotiate rates do so in the light of the public benchmarks. Hospitals wield considerable pricing power with respect to private insurers, and it is here that the antitrust critique is at its strongest. But even here the solution may not lie in antitrust enforcement on the supply side of health care markets but in more sophisticated purchasing on the demand side. Some insurers have experimented with narrow contractual networks, excluding providers charging the highest rates, but find that employers and employees often insist on broad networks and unconstrained choice. Some employers have experimented with reference pricing in benefit design, where payment is capped at the level of the affordable options, but find that employees are reluctant to pay the difference when selecting a more expensive test or treatment. Observing little reward in volume gains for lower prices, providers faced with rising costs institute another round of price increases.

#### What About Innovation?

Some products and services are characterized by rapid rates of technological innovation, innovation that commands a decisive cost or quality advantage and that strikes not at the margins of the profits and the outputs of the existing firms but at their foundations and their very lives. As evident in biopharmaceuticals, medical devices, and molecular diagnostics, innovation thrives in an ecosystem that includes both entrepreneurial startups with close links to research universities and diversified conglomerates with expertise in manufacturing and distribution. The antitrust tradition tends to view health care markets as stable and secure, dominated by large organizations that exploit their market positions to gouge consumers and patients. But many markets are unstable and turbulent, competitive and contestable, a war of all against all. Health care competition is in part neoclassical in nature, with similar organizations pursuing incremental improvements in efficiency and quality, but also Schumpeterian, subject to disruption by new clinical technologies and the new facilities, professions, and pathways that accommodate them.

## A New Vision

Antitrust policy has been ineffective in preventing the growth of health care organizations and the consolidation of health care markets, and its prospects for the future are less than compelling. Many health care organizations already are large, albeit diversified. Many health care markets already are consolidated, albeit contestable. Perhaps the greatest challenge facing antitrust policy in health care has been its failure to articulate its vision for the socially desirable structure of organizations and markets.

What do antitrust economists and regulators want? Do they seek a return to the cottage industry which, it should be noted, remains alive and well in many corners of the health care system? Are small and single-specialty medical practices more efficient than large multispecialty medical groups? Do independent community hospitals offer lower costs and higher quality than regional chains? Is innovation most rapid in a cottage industry or one that combines startups with global champions? Do we want to backtrack from value-based care that is coordinated across clinical specialties, facilities, and populations? Do we want patients to shop the market when they are sick, each time they need a physician visit, diagnostic test, surgical procedure, or hospital admission, or do we want them to shop among integrated organizations when they are well?

Some critics of integrated organization acknowledge the failings of the cottage industry but favor ‘focused factories’ that pursue economies of scale in particular procedures rather than economies of scope across the care continuum. Examples include hospitals specialized in cardiac care, orthopedic surgery, and other well-paid specialties. But these narrowly structured organizations have been confounded by the fact that many patients with severe clinical needs have comorbid conditions and cannot be parsed out to one specialty provider.

## A New Humility

None of this discussion should not be construed as support for overbuilt health care conglomerates and innovation-resistant health care markets. An overbuilt organization is hard to define but, like pornography, you know it when you see it. In a competitive market, they would be rightsized by divestitures or the discipline of bankruptcy. In the highly regulated health care market, they may stagger on, burning through their endowments and shareholder equity. And ditto for an innovation-resistant market. If parts of health care continually are buffeted by the gale of creative destruction, others proceed decade after decade with many of the same providers, professions, and processes.

Competition is important to the extent it promotes coordination, since coordination is the key to efficiency and innovation. The question is how to coordinate, not whether to coordinate. The revolution in digital technology and artificial intelligence evokes the question of whether this coordination can be achieved without organizational integration. For some products and services this will certainly be the case. Novel forms of contract can substitute for internal organization. But advances in information technology often complement rather than replace formal organization to identify new ways of doing what needs to be done.

An honest assessment suggests that many organizational and market structures perform well, at least for some products. In our ignorance, let us fall back on the market test and not be overconfident as to our ability, and that of the antitrust regulators, to identify which structures should thrive and which should not. While there is competition between organizations, there also is competition between organizational forms. The market for organizational forms has proven relatively efficient, over time, in marginalizing structures that once were adapted to their environment but did not adjust well when their environment changed. Market contracts, organizational hierarchies, and hybrid forms all have much to offer. Let antitrust policy adopt its own Hippocratic Oath. Above all else, do no direct harm.

